# Letter from T. G. Williams, M.D., Watertown

**Published:** 1871-06

**Authors:** F. G. Williams

**Affiliations:** Watertown, Wis.


### Correspondence.

WATERTOWN, WIS., *April 11, 1871.*

DEAR EXAMINER,—It has been my intention for some time to send my experience in the use of the "Bromides in Summer Complaints of Children." It was my habit to give the brom. potass in from $\frac{1}{2}$ to 2 gr. doses every one, two, or three hours, according to age and symptoms. At first, however, when called to a very severe case my courage would fail me, though I had found no occasion for it, and I would resort to the usual treatment. After reading Dr. Caro's report on the use of the "Brom. Potass. in Summer Complaints of Children," in the August number of the EXAMINER, for 1869, I had more confidence in the treatment. This report agreed so nearly with my own views of the physiological action of the bromides that I had but little hesitation in accepting his conclusions. After a faithful trial, during two seasons in which there has been a great prevalence of diarrhœa and dysentery, I am almost as confident of the value of the treatment as is Dr. Caro. Though I could not report as many cases I could report some as remarkable. I have that confidence in the treatment that I do not despair so long as life remains. I have been called to cases where it seemed useless to do anything, but would leave medicine and orders to let me know if child were living next day. Would always have occasion to repeat my visit, and find decided improvement. Have had but two or three deaths since I have relied on this course of treatment, and they were scrofulous children. I am aware that remedies which have in the practice of some been very popular have wholly failed with others. At present I have but few remedies in which I place so much confidence as in this form of treatment. At first I used the bromide alone in simple solution, gr. xx. to grs. xxx. to the ℥j. I am now in the habit of using syrp. rhubarb, vil., as menstruum. ℞. Syrp. rhubarb, vil. ℥ij., potass. brom. ℈ij., ♏., s. gtt. x. to xx., every one, two, or three hours. If there is much pain or restlessness I add to above from ½ to 1 gr. mor. sulph. After further trial I may lose some of my present confidence. I would like to hear from others on this subject, for it is only by comparing views we can ever reach anything of certainty in therapeutics.

The necessary precautions for nursing should always be strictly urged. Bathing, pure air, prudent clothing, and a rigid attention to the diet, which should be fresh milk in small quantities, are all important. These without medicine would answer a better purpose than medicine with inattention to these particulars. Many more of these little sufferers would be spared if we should take more pains in giving directions for nursing. So many nurses are to be found who think that if they give the medicine to the minute, as the doctor ordered, they are doing their whole duty.

